# 

**Published:** 1892-06

**Authors:** 


### CONTENTS FOR JUNE, 1892.

ORIGINAL COMMUNICATIONS. PAGE.

Diagnosis and Treatment of Abscess of the Ano-Rectal Region. By EDWARD CLARK, M. D. 641
Does Organic Disease of the Heart Preclude the Use of Chloroform in Parturition? By T. RIDGWAY BARKER, M. D. 649
Inert Tubercle Bacilli—Other More Dangerous Organisms in the Sputum and Lungs. By F. J. THORNBURY, M. D. 654

SOCIETY PROCEEDINGS.

Philadelphia County Medical Society 656
New York Academy of Medicine—Section on Orthopedic Surgery 665

SELECTIONS AND ABSTRACTS.

The Gynecological Work of Dr. Joseph Price 676

EDITORIAL.

The College Commencements 679
Topics of the Month 681

PERSONAL.

Dr. J. B. Andrews—Dr. Gilbert I. Cullen—Dr. M. D. Mann 689

OBITUARY.

Dr. James Ross—Mr. Orsamus G. Warren 690

SOCIETY MEETINGS.

The Eleventh International Medical Congress—The American Academy of Medicine—The American Pharmaceutical Association—The Medical Society of the State of New York—The American Electro-Therapeutic Association—The American Association of Obstetricans and Gynecologists—The Mississippi Valley Medical Association 691
The Medical Society of the County of Erie 692

BOOK REVIEWS.

A Manual of Operative Surgery; Treves 692
Treatise on Gynecology. Medical and Surgical; Pozzi 695
The International Medical Annual and Practitioners' Index for 1892 696
The Mütter Lectures on Surgical Pathology 697
Consumption: How to Prevent it and How to Live with It.—Saunders' Pocket Medical Formulary 698
Essentials of Physics—Lehrbuch der Hebammenkunst—Lessons in the Diagnosis and Treatment of Eye Diseases 699
Deafness and Discharges from the Ear—A Dictionary of Treatment, or Therapeutic Index—Cancer and its Treatment 700
Books Received 701
Miscellany 703

